# Leveraging Dog Models to Uncover Human Cancer Insights

**DOI:** 10.21203/rs.3.rs-9783746/v1

**Published:** 2026-05-28

**Authors:** Geesa Daluwatumulle, Leslie A. Smith, Nathan Glen, Ji-Hyun Lee, Nathan D. Seligson, James A. Cahill, Kiley Graim

**Affiliations:** 1 Department of Computer & Information Science & Engineering, University of Florida, 1889 Museum Road, Gainesville, 32611, FL, USA.; 2 Environmental Engineering Sciences Department, University of Florida, 1949 Stadium Road, Gainesville, 32611, FL, USA.; 3 University of Florida Genetics Institute, University of Florida, 2033 Mowry Road, Gainesville, 32610, FL, USA.; 4 University of Florida Health Cancer Institute, University of Florida, 2033 Mowry Road, Gainesville, 32610, FL, USA.; 5 Department of Biostatistics, University of Florida, 2004 Mowry Road, Gainesville, 32611, FL, USA.; 6 Department of Pharmacotherapy & Translational Research, University of Florida, 1225 Center Drive, Gainesville, 32610, FL, USA.; 7 Nemours Children’s Health, Jacksonville, FL, USA.

**Keywords:** comparative transcriptomics, dog model, adult, pediatric, cancer, RNA-seq

## Abstract

**Background:**

Comparative genomics can reveal insights into human disease that cannot be identified from human data alone. Dogs are particularly useful for comparative genomics studies as they share human diet and environment, and, from a genomics perspective, dogs are closely related to humans. Dogs develop spontaneous tumors that closely resemble human tumors, have a high tumor incidence rate, and their popularity as pets ensures the wide availability of samples for scientific studies. Despite these benefits, no pancancer comparative transcriptomic study of human and dog cancers exists, nor has any study systematically quantified the effectiveness of dog tumors as a model of human adult and pediatric cancers. Individual cancer studies reveal both similarities and differences between species, but the extent of the molecular similarity across tumor types remains unclear.

**Methods:**

To address this gap, we performed a pancancer analysis of 5,875 samples (913 dogs and 4,962 human samples, including 806 pediatric samples) spanning 11 tumor types. We developed a formula to quantify transcriptomic similarities between human and dog cancers across several metrics.

**Results:**

We show that, overall, dogs are an excellent model for many human cancers and that dogs tend to be better models of adult cancers than pediatric cancers, with notable exceptions such as gliomas and sarcomas.

**Conclusions:**

Our scalable approach enables rapid and accurate identification of model systems for studying human cancers, creating new opportunities for comparative oncology studies across the tree of life.

## 1 Background

Given the low success rates in translating preclinical discoveries into human cancer treatments [1–4], it is increasingly essential to consider alternative approaches to cancer research. One promising avenue is comparative oncology. Animals with naturally occurring tumors, such as dogs, have gained increasing attention in translational medicine [2, 5–8]. Dogs tumors parallel human tumors with respect to histological, clinical, and molecular characteristics [2, 6, 9–14]. They share human diet and environment [15, 16] and naturally develop tumors at incidence rates comparable to humans [3]. Certain cancers that are rare in humans, such as osteosarcomas, occur more frequently in dogs, making them a valuable resource for studying rare cancers where large human cohorts are difficult to recruit [2, 4, 17, 18]. Dog develops tumors naturally and have an active immune environment and tumor microenvironment [19–21]. Additionally, the presence of an intact immune system and tumor heterogeneity makes dog genomics studies a powerful complement to studies in mice, where tumors are often induced and may lack the complexity of naturally occurring cancers. Altogether, these factors render dogs a uniquely powerful resource for studying human cancer.

Previous studies have evaluated molecular similarities between dog and human tumors from various cancer types. For example, comparative genomics studies have identified parallels between human and dog in breast cancer [10, 22–24], sarcoma [25–30], pediatric osteosarcoma [31–35], bladder cancer [36–38, 38–44], adult/pediatric glioma [45, 46], lymphoma [11, 47–51], and melanoma [52–57]. Observations from dog clinical trials have provided valuable insights for pediatric and adult osteosarcomas expressing the tyrosine kinase receptor HER2/neu [34] and informed the use of COX inhibitors in the treatment of urinary and bladder cancers [2, 58, 59]. Favorable outcomes discovered as part of a DNA vaccine for dog melanoma influenced a phase 1 trial in human melanoma patients [60, 61]. Dog studies are used to evaluate toxicity profiles [62–65] and to determine dose and regimen in several cancer therapeutics [2, 66]. The U.S. National Academy of Medicine’s National Cancer Policy Forum organized a 2016 workshop which highlighted the value of dog cancers [3]. These examples show the broader potential of dog cancers as valuable translational models for human cancers.

Dog tumors, however, are not universally strong models of human tumors. Differences between dog and human tumors have been described in genomic profiles in thyroid carcinoma [67], histological and morphological characteristics in prostate cancer [9], regulatory T cell expression in hepatocellular carcinoma [68], levels of aneuploidy in adult gliomas [46], the frequency of gains and losses in leukemia subtypes [69], and *CDKN2A* expression in lymphomas [70]. Additionally, many lack a rigorous scientific evaluation of the molecular parallels between dog and human tumors, including head and neck cancer [71, 72], leukemia, prostate cancer [9, 73, 74] and adrenocortical carcinoma [75]. Given these knowledge gaps, the extent to which a particular dog cancer faithfully model its human counterpart remains unclear, and no pancancer transcriptomic analysis of human and dog tumors exist to date.

Here, we develop a quantitative framework to assess the effectiveness of dogs as translational models of human adult and pediatric cancers. We apply this framework to a pancancer compendium of dog, human adult, and human pediatric tumors. Results from this framework recapitulate previous findings, correctly identifying positive and negative controls as strong and weak models, respectively, of their human tumor counterparts. We also apply our formula to cancers that are not as well studied, quantifying their robustness as a model of human cancer. Across all cancers, we find common patterns of species-specificity and quantify their impacts in comparative cancer modeling. Our multi-dimensional formula incorporates these and cancer-specific information to provide a single score of model robustness. Critically, we identify that clustering metrics better describe similarities between transcriptomic profiles of dog and human tumors than previously applied differential expression approaches, as they take into account tumor type heterogeneity. Altogether, we provide a quantitative computational strategy to identify robust models of human cancers.

## 2 Methods

All analyses here were performed using the R programming language (version 4.5). Unless otherwise indicated, human adult and pediatric tumors are not compared or grouped together in any analysis.

### 2.1 Creating a multi-species pancancer compendium

We created a multi-species pancancer compendium by harmonizing samples from three publicly available sources. Dog samples originate from 45 original studies compiled by Cahill et al. [5], adult from The Cancer Genome Atlas (TCGA) [76], and pediatric from the Treehouse Childhood Cancer Initiative (Treehouse) [77]. From all datasets, we included only primary tumors. Benign tumors, samples with low expression counts (dog only; bottom 1% of samples), pediatric TCGA samples (AGE ≤ 18), and adult Treehouse samples (AGE > 18) were excluded from our analysis.

TCGA samples were downloaded as batch-normalized RNA-seq RSEM counts. Dog and pediatric datasets were downloaded as trimmed mean of M-values (TMM), which we selected because this approach most closely matched TCGA data. We corrected for batch effects across studies within species and after combining species datasets using Combat [78]. Next, we mapped dog genes to their human orthologs using Orthogene [79] with one-to-many mapping, and Ensembl genes to HUGO gene names for the pediatric dataset using BioMart [80]. After ortholog mapping, we removed duplicated genes by reducing to the median value across duplicates. Lastly, we log-transformed each dataset and combined the three datasets into one joint dataset, based on orthologous genes.

We next harmonized cancer type labels across species (Supplemental Table 3). We filtered adult melanoma samples to include only skin cutaneous melanoma and soft tissue sarcoma from dogs. After cancer type label harmonization, cancers with <10 samples per species were removed from the dataset. Ten adult and six pediatric cancers met this criteria, totaling eleven unique cancer types. After harmonization and quality control, the resultant dataset included 12,171 genes and 5,875 samples spanning 11 cancers (see Table 1). This harmonized dataset is used for all subsequent analysis.

### 2.2 High-level data exploration

**Fig. 1:**
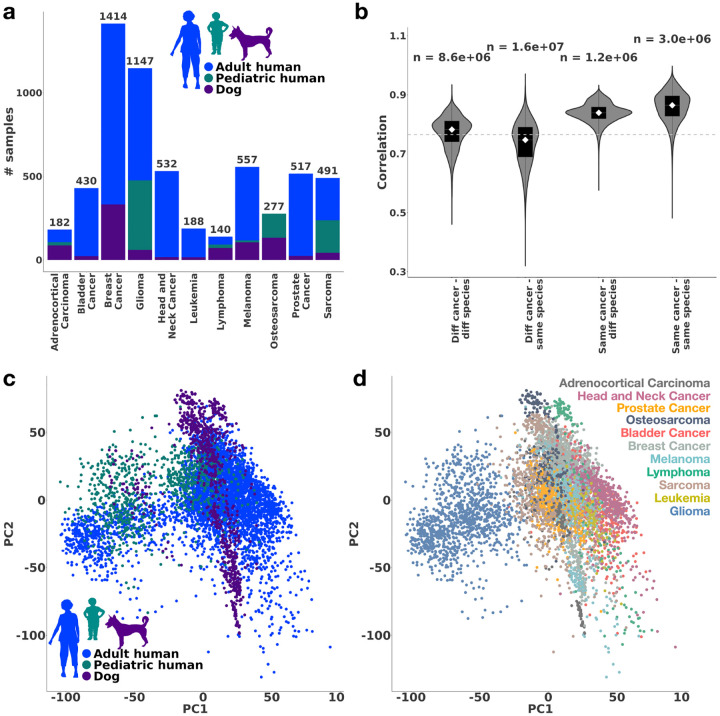
The human and dog pancancer compendium **(a)** Distribution of cancer types in the dataset, showing the number of samples per cancer type and species. **(b)** Sample-sample correlations show that the cancer type effect is stronger than the species effect, where the mean difference was 0.1007 (95% CI: 0.10067–0.10081) for diff cancer-same species and same cancer-diff species comparison. Adult-pediatric correlations were excluded from this plot and dashed line shows the average correlation and the white diamond shows the median correlation for that group. The total number of sample-sample pairs are annotated at the top. **(c-d)** PCA of all samples, color-coded by **(c)** species and **(d)** cancer type. Each dot represents a sample.

Exploratory data analysis (EDA) was conducted in two phases. In the first phase, we performed EDA on the entire dataset to understand the data distribution and identify cancer-type patterns using principal component analysis (PCA) (see Figure 1c, d). In the second phase, we performed EDA for each cancer type listed in Table 1 to uncover overall molecular-level characteristics unique to each cancer by calculating the median gene expression levels across human and dog samples.

In the first phase, we calculated pairwise sample correlations and categorized each sample pair into four groups based on whether the samples shared the same cancer type and/or species. Statistical differences between the four groups were evaluated using bootstrap resampling (500 replicates, percentile method) to construct 95% confidence intervals (CI) for the difference in means between each pair of groups. We applied the same approach to inter-species (*adult-dog* and *pediatric-dog*) and intra-species comparisons (*dog-dog*, *adult-adult*, and *pediatric-pediatric*) (Supplemental Figure 2a), as well as to the heterogeneity analysis (Supplemental Figure 6a), using 1,000 replicates. Additionally, we tested whether samples largely group by cancer type in the PCA space by calculating distances between pairs of samples using PC1 and PC2 and grouping them into within (same cancer) and between cancer types (different cancer) and performing a one-sided Wilcoxon rank sum test.

### 2.3 Differential expression and enrichment analysis

For each cancer type, we performed differential expression analysis between human and dog tumor samples using DESeq2 [81]. We used an adjusted P-value of 0.05 to identify genes differentially expressed (DEGs) in human and dog tumors, then performed Gene Ontology (GO) biological processes (BP) enrichment analysis using topGO [82] to find functional differences between species that may impact cancer analysis. GO terms with fewer than 10 genes were not considered. We also calculated enrichment of Catalogue of Somatic Mutations in Cancer (COSMIC) [83] cancer genes (see Additional file 1) and the COSMIC Hallmarks of Cancer gene set in the differentially expressed genes. To do this, we generated a .gmt file from the COSMIC Hallmarks dataset and used clusterProfiler [84] for functional enrichment analysis. Using the same package, we calculated the molecular signatures database (MSigDB) C6 oncogenic gene set enrichment [85, 86]. For all enrichment analyses, we used an adjusted P-value threshold of 0.05 while keeping other configurations at their default settings. We used GOSemSim [87] to rank the two sets of GO BPs by semantic similarity (Figure 5a).

We performed binomial tests with the Benjamini-Hochberg (BH) correction to determine whether cancer-specific enrichment of DEGs in each tumor type was greater than expected by chance. Additionally, we tested whether annotation bias in the COSMIC database influenced downstream results. We used the grouped somatic tumor types, calculated the number of genes in each category, binarized category size using the median number of genes as a threshold, and performed a Fisher’s exact test comparing model robustness and COSMIC category size (see Additional file 1).

### 2.4 Clustering

#### 2.4.1 Species-specific clustering

We clustered five distinct subsets of the data (adult only, pediatric only, dog only, adult and dog, pediatric and dog) on each tumor type using hierarchical clustering within the ConsensusClusterPlus [88] package. We evaluated clustering performance based on the cumulative distribution function (CDF) curve and the relative change in area under the CDF curve (ΔK) to identify the optimal number of clusters by locating the point just before the ΔK plateau. We then applied the optimal *k* from the human-only analysis to cluster dog samples. For all five clustering analyses, we set the number of subsampling iterations (reps) to 1,000. We used repeated clustering with subsampling with the default configuration parameters (pItem = 0.8) in both the human- and dog-only clustering steps. On the combined clustering, we set the proportion of items sampled (pItem) to ensure equal representation of human and dog samples as there were far more human samples than dog. We also adjusted the item sampling weights (weightsItem) to keep all samples from the minority species and subsample the majority species by setting the minority group sample weights to 1 and the rest to 0.5.

To calculate cluster enrichment, we performed Fisher’s exact test with simulated P-value on (1) adult-only clustering and TCGA subtype labels (2) dog-only clustering and combined species clustering, and (3) human-only clustering and combined species clustering and adjusted for multiple testing correction using BH. Here, human can be either adult or pediatric and combined can be either adult and dog or pediatric and dog. For TCGA subtypes, we considered breast cancer (BRCA) subtypes Basal, HER2, LumA, and LumB; glioma (GBM/LGG) subtypes GBM and LGG; head and neck cancer (HNSC) subtypes HPV− and HPV+; and sarcoma (SARC) subtypes DDLPS, LMS, MFS/UPS, and Other. The HPV+ subtype was retained as a reference to validate model performance in cross-species comparisons.

#### 2.4.2 Organ system clustering

To assess whether organ system clusters identified in TCGA [76, 89, 90] are also present in dog cancers, we performed hierarchical clustering on combined dog and adult data for BRCA, HNSC, and bladder cancer (BLCA). We grouped BRCA and HNSC as pan-squamous and BRCA as pan-GYN to define organ system-level categories. We selected the top 5,000 most variable genes, applied z-score normalization, then used the clustering approach described in Section 2.4.1. We performed BH-adjusted Fisher’s exact tests on cluster groups and organ systems to see if each species recapitulate organ system patterns mentioned in the TCGA pancancer analysis.

### 2.5 Predictive modeling

#### 2.5.1 Tumor subtype classification

We trained decision tree, random forest, and elastic net models for each cancer type (BRCA, GBM/LGG, HNSC, and SARC) to classify TCGA subtypes using the R tidymodels package [91]. Adult tumor samples were split into two datasets stratified by subtype, train ($\frac{2}{3}$) and test ($\frac{1}{3}$), and model parameters were optimized on the train dataset. We tuned the hyperparameters of each model using k-fold cross-validation and evaluated performance using area under the curve (AUC). To address class imbalance in the data, we weighted samples inversely to their class frequencies and included these weights into model training. The best-performing model was then applied to dog samples to assign subtypes.

For all dog samples, we validated the model by testing if the mean predicted probability was higher than the random chance threshold ($\frac{1}{k}$, where *k* is the number of subtypes). To determine whether the model captured meaningful subtype distinctions, we compared sample-sample similarity for pairs with the same predicted subtype and pairs with different predicted subtypes within each cancer type (BRCA, GBM/LGG, HNSC, and SARC). Pairwise similarity was quantified as the absolute difference in predicted probabilities. We used a bootstrap resampling (1,000 replicates, percentile method) to construct 95% CI and to evaluate whether the differences between groups were statistically significant.

#### 2.5.2 Cancer type classification

We next calculated predictive performance across all tumor types to quantify the similarity of dog cancers to human cancers. For this analysis, we used the entire human cancer dataset for training and dog samples as the test set. As before, we applied class weights in the training data to address class imbalance in the dataset, then tuned and evaluated the models using the same approaches discussed in the tumor subtype classification task.

### 2.6 A quantitative formula for model robustness

Lastly, we combine the outputs of previous analyses plus several new factors, described below, into a quantitative formula for model robustness. These factors all provide unique perspectives of similarities between species’ data. Together, they provide diverse but comprehensive insights into cross-species similarities and differences.

#### 2.6.1 Integrating overall patterns of expression

We used the median gene expression (M) calculated across humans and dogs for each tumor type in the EDA phase. We then calculated the Wasserstein distance (W) between these values to quantify inter-species differences in median gene expression. Finally, we normalized this using the global expression range of all cancer types, we denote this feature as EDA_1. Equation 1 shows the formula for EDA_1, which captures the normalized absolute distance between inter-species median gene expression values for each cancer.


(1) $$\text{EDA}\_1=\frac{\text{W}\left(M_{\text{human}},M_{\text{dog}}\right)}{\text{Max}\left(M_{\text{all}}\right)-\text{Min}\left(M_{\text{all}}\right)}$$

We grouped the previously calculated correlations (corr) in the EDA phase into inter-species and intra-species, and ranked cancer types based on how close the median (med) inter-species correlations are to the mean intra-species median correlations, defined as EDA_2. Equation 2 shows the formula used to make this ranking. In both formulas, human can be either adult or pediatric but not both.


(2) $$\text{EDA}\_2=\frac{\text{med}\left({\text{Corr}}_{\text{human-dog}}\right)}{\frac{\text{med}\left({\text{Corr}}_{\text{dog-dog}}\right)+\text{med}\left({\text{Corr}}_{\text{human-human}}\right)}{2}}$$

#### 2.6.2 Integrating enrichment analysis

Using the COSMIC gene enrichment results from the differential expression analysis, we calculated the fraction of cancer-related genes among all COSMIC genes identified within the differentially expressed genes. This was repeated using the MSigDB C6 enrichment to calculate the proportion of uniquely enriched terms relative to the total number of terms in the gene set. These are represented by DEA_1 and DEA_2, respectively, in Equation (3).

#### 2.6.3 Integrating supervised and unsupervised model performance

To create a single score of the effectiveness of the supervised and unsupervised modeling at identifying cancer-specific subtypes, we used the combined species clustering and calculated a score based on inter-species distances within the first two principal components. Specifically, we calculated species-cluster centroids based on the mean PC1 and PC2 values. We then computed pairwise Euclidean distances between centroids, normalized them to the observed minimum and maximum distances, and extracted the distances between human and dog centroids. To account for differences in the proportion of dog tumors assigned to each cluster, we weighted these distances by the number of dog samples per cluster. We defined the inter-species similarity score as the minimum weighted human-dog centroid distance, thereby ensuring that even a subset of dog samples clustering closely with human cancers contributed to the score. This score is referred to as Clustering in the below sections. In addition to these unsupervised analysis comparisons, we used the predicted probabilities from the cancer type prediction task and calculated the average probability for samples that were correctly classified in the dog test set and ranked all cancers, hereafter referred to as the ML feature.

#### 2.6.4 A quantitative formula

Using the above methodological approaches (EDA_1, EDA_2, DEA_1, DEA_2, Clustering, ML), we created a formula to quantify how strongly a given dog cancer recapitulates a human cancer (Equation (3)). For the EDA_1, DEA_1, DEA_2, and Clustering, lower values indicate better performance, so we transformed those as described in Equation (3). We then estimated the weights for each feature using a subset of the adult tumor data; BRCA, leukemia (LAML), BLCA, GBM/LGG, adrenocortical carcinoma (ACC), melanoma (SKCM), and lymphoma (DLBC), labeling suitability as 0 for LAML and ACC, and 1 for all other tumor types [22, 41, 46, 53, 75, 92, 93]. We included LAML as a negative control because it represents different tumor types between adult and dog. ACC was also included as a negative control because, although ACC shares some molecular features, dog tumors form distinct transcriptomic groups that do not fully align with the classical human adrenocortical adenoma and ACC classification [75]. We calculated each weight using the differences in medians between the two groups (Δ median) and applied the resulting formula *f*(*x*_*i*_) to both adult and pediatric cancers. The score ranges from 0 to 1, where 1 indicates perfect similarity.


(3) $$\text{Score}=\sum_{i=1}^{6}w_{i}f\left(x_{i}\right),\;f\left(x_{i}\right)=\left\{\begin{array}{ll} \frac{1-x_{i}}{1+x_{i}}, & i=1,3,4,5\\ x_{i}, & i=2,6 \end{array}\right.$$

The input *x*_*i*_ correspond to the following methods and the weights are indicated by *w*_*i*_:

$$x_{1}=\text{EDA}\_1\;x_{2}=\text{EDA}\_2\;x_{3}=\text{DEA}\_1\;x_{4}=\text{DEA}\_2\;x_{5}=\text{Clustering}\;x_{6}=\text{ML}$$

## 3 Results

To assess the molecular similarities between dog and human cancers at the broader-level, we created and analyzed a pancancer dataset containing samples from 11 dog, 10 human adult, and 6 human pediatric cancers. Samples from Cahill et al. [5], TCGA [76], and Treehouse [77] were combined into one harmonized dataset containing 5,875 tumor samples and 12,171 human-dog orthologous genes (Figure 1a; Table 1). All analyses in this study were performed on this joint dataset. We create a formula of overall molecular similarity across multiple molecular perspectives.

We began by quantifying transcriptome-wide gene expression similarities between dog and human samples and found that, within each cancer type, per-gene median expression is similar across species (normalized absolute distances 0.007 – 0.047; Supplemental Figure 1a). PCA plots in Figure 1c–d visually demonstrate that the harmonized multi-species data group by tumor type rather than species. We performed bootstrap 95% CI to evaluate whether there are statistically significant differences between cancer types and species, excluding pairs of samples where one sample is adult and the other is pediatric. From this analysis we found that the cancer type effect is stronger than the species effect; the diff cancer-same species group differed significantly from the same cancer-diff species group (Figure 1b), with a mean difference of 0.1007 (95% CI: 0.10067–0.10081). Other comparisons showed smaller but significant differences between the same cancer-same species and same cancer-diff species groups (mean difference 0.0215, 95% CI: 0.02146–0.02162), and between the same cancer-diff species and diff cancer-diff species groups (0.0646, 95% CI: 0.06454–0.06467).

Using the previously calculated sample-sample Pearson correlations, we next compared pairs of samples from the same cancer and species (intra-species) to pairs from the same cancer but different species (inter-species). For every pairwise group comparison, the bootstrap 95% CI for mean difference excluded zero (Supplemental Figure 2a, Supplemental Table 2). However, mean inter-species correlations were lower than mean intra-species correlations for all cancers except adult BLCA and adult/pediatric SARC. Given all comparisons were statistically significant, we quantified the relative similarity between inter- and intra-species correlations using the ratio defined in Equation 2 and found that, among adult cancers, BRCA showed the highest ratio, followed by SKCM and HNSC (Supplemental Table 1). In pediatric cancers, GBM/LGG and osteosarcoma (OS) exhibited the highest ratio (Supplemental Table 1). ACC had the lowest ratio for both adult and pediatric cancers.

### 3.1 Gene expression differences between species are mostly not cancer related

**Fig. 2:**
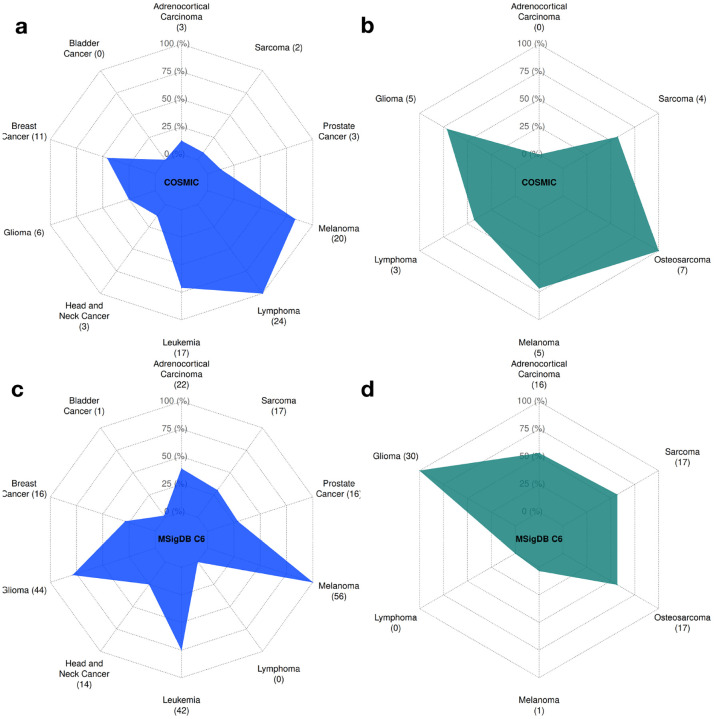
Differential expression analysis between dog and human cancers and gene association with COSMIC and MSigDB. We analyzed differentially expressed genes using 763 COSMIC genes and 189 unique MSigDB C6 oncogenic signatures. Radial plots show the percentage of **(a-b)** COSMIC cancer genes and the enriched cellular pathways from **(c-d)** MSigDB identified through dog vs human differential expression analyses for each cancer type in adult and pediatric samples. The corresponding number of terms are annotated in the labels; adult (left), pediatric (right). For COSMIC, the annotations reflect the cancer-related genes that are differentially expressed between species. In the adult vs dog analysis, the total number of unique COSMIC genes differentially expressed is: adrenocortical carcinoma (n=278), bladder (n=25), brain (n=59), breast (n=185), head and neck (n=13), leukemia (n=69), lymphoma (n=171), melanoma (n=84), prostate (n=37), and sarcoma (n=44) and in pediatric vs dog analysis is: adrenocortical carcinoma (n=114), brain (n=36), lymphoma (n=11), melanoma (n=10), osteosarcoma (n=64) and sarcoma (n=26). In this context, smaller number of differentially expressed COSMIC genes reflect fewer dysregulated cancer-associated processes and therefore suggest greater molecular similarity between species.

To quantify the degree to which transcriptomic differences between human and dog tumors are cancer related, we ran differential expression analysis on each cancer type comparing human and dog samples to identify DEGs (adult vs dog, pediatric vs dog). We performed gene set enrichment analysis on these DEGs to identify differentially expressed biological processes. Figure 2a–d shows enrichment of COSMIC cancer-related genes and MSigDB C6 oncogenic signatures across all cancers. BLCA had the lowest percentage of cancer-specific DEGs (0%) among the 10 adult cancers, and DLBC the highest (24%). Both are considered positive controls in this study, highlighting the importance of using multiple metrics of similarity. In the 6 pediatric cancers, ACC had the lowest percentage of cancer-specific DEGs (0%) and OS had the highest (7%). The binomial test gave significant enrichment for cancer-specific genes for pediatric GBM/LGG (one-sided binomial-test; BH adjusted *P*-value = 2.70 × 10^−2^), SKCM (one-sided binomial-test; BH adjusted *P*-value = 6.72 × 10^−4^), OS (one-sided binomial-test; BH adjusted *P*-value = 1.60 × 10^−2^), and SARC (one-sided binomial-test; BH adjusted *P*-value = 1.73 × 10^−2^). ACC, one of the negative controls, was the only cancer with any enrichment of COSMIC Hallmarks of Cancer genes. Among adult cancers, the number of dysregulated C6 cellular pathways was lowest in DLBC and highest in SKCM, while in pediatric cancers, again DLBC showed the lowest and GBM/LGG showed the highest pathway dysregulation (Figure 2c–d). Overall enrichment of cancer-related pathways is similar in adult versus pediatric cancers (median 16.5 enriched terms for both adult and pediatric), however there is significant variability.

### 3.2 Tumor subtypes span species

Dogs present with clinically-actionable breast cancer molecular subtypes defined in human studies [22, 94, 95]. To determine if dogs also share human cancer subtypes in other cancers, we calculated statistical enrichment between the clustering solutions and clinical subtypes (see Methods). As a baseline, we compared our adult-only clustering results with previously published TCGA subtypes (BRCA, GBM/LGG, HNSC, SARC) and found a statistically significant correlation between the two (Fisher’s exact test: BH adjusted *P*-value = 4.9975 × 10^−04^; Supplemental Figure 3a). Overlap between dog-only cluster labels and adult-dog cluster labels were statistically significant for HNSC, SKCM, BRCA, SARC, DLBC, ACC (Fisher’s exact test: BH adjusted *P*-value = 5.830 × 10^−04^), and BLCA (Fisher’s exact test: BH adjusted *P*-value= 5.497 × 10^−03^). All adult-only and adult-dog enrichments were significant (Fisher’s exact test: BH adjusted *P*-value= 4.9975 × 10^−04^). Dog-only and pediatric-dog clustering solutions statistically correlate for OS, SKCM, SARC, ACC (Fisher’s exact test: BH adjusted *P*-value= 4.998 × 10^−04^) and DLBC (Fisher’s exact test: BH adjusted *P*-value= 4.860 × 10^−06^). Pediatric-only and pediatric-dog enrichment in GBM/LGG and SARC were also significant (Fisher’s exact test: BH adjusted *P-*value= 4.998 × 10^−04^). We excluded cancers with insufficient cluster variability from enrichment testing. The combined species clustering is visualized in Supplemental Figure 4a.

Next we used the minimum weighted human-dog centroid distance (see Methods) to quantify the distribution of sample overlap across all cancers. A small distance indicates that there is considerable overlap between samples from both species. This scoring was applied to each cancer. We found that, among adult cancers, BLCA followed by SKCM, HNSC, and GBM/LGG showed the smallest distances. ACC and LAML, our negative controls, exhibited the greatest separation between dog and adult cancers. In pediatric cancers, GBM/LGG had the smallest distance and ACC the largest (Supplemental Table 1).

### 3.3 Predictive modeling identifies conservation across species

Comparative genomics models do not necessarily have a one-to-one correspondence between tissue type and tumor type. A tumor from a model organism may be a robust model of a human tumor in a different tissue type. As an alternative approach to investigate the robustness of the tumor types across species, we trained several machine learning algorithms to predict tumor type within the human data. We selected optimal models for each adult and pediatric cancers based on AUC. In adult samples, the elastic net model had the highest performance (AUC 0.92) and for pediatric samples, the random forest model was the best model (AUC 0.99). These models were applied to dog samples (Supplemental Figure 5a) and we calculated the average predicted probability over the dog samples that were correctly classified (Supplemental Table 1). Among adult cancers, GBM/LGG had the highest average probability, followed by SKCM. ACC and prostate cancer (PRAD) had the lowest. In pediatric cancers, GBM/LGG and SARC showed the highest probabilities, and ACC the lowest.

**Fig. 3:**
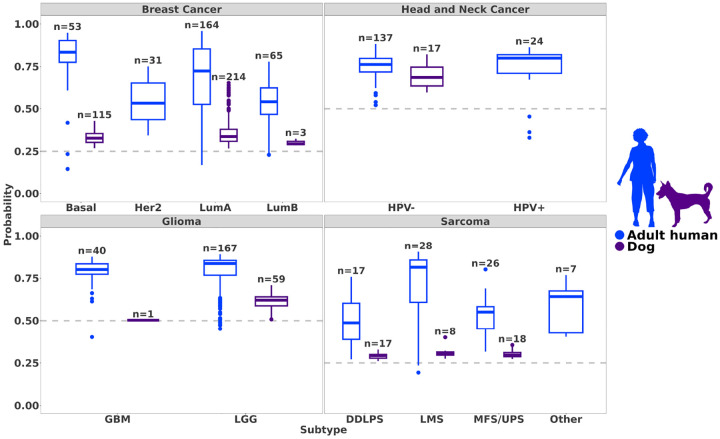
Cross-species classification reveals transcriptomic similarities. Box plots showing predicted subtype probabilities after training individual classifiers on adult cancers with subtype data and applying it to human and dog held out test sets. Dotted gray lines indicate random chance, defined as $\frac{1}{k}$, where k is the number of subtypes. The y-axis represents the predicted probability of the sample. Specifically, the adult probabilities represent the probability values of the true subtype label irrespective of the predicted class. As expected, we didn’t see any predicted HPV+ subtype in dogs.

Previous studies [94, 96] have noted that several human tumor types have clinically-actionable subtypes that may confound analysis. Additionally, dog tumors may only accurately recapitulate one or more of the human subtypes. To evaluate these factors, we trained machine learning models on the four adult cancers with TCGA subtypes (BRCA, GBM/LGG, HNSC, SARC; Figure 3a). Models were trained on each tumor type independently. We performed bootstrap 95% CI and found that the models captured meaningful subtype distinctions for all four cancers (see Methods). The differences in mean similarity were −0.0072 (95% CI: −0.0080 to −0.0064) for BRCA, 0.0662 (0.0587 to 0.0733) for GBM/LGG, and 0.0042 (0.0019 to 0.0065) for SARC, with all confidence intervals excluding zero. HNSC was not included as only a single class was predicted.

### 3.4 A formula quantifying tumor-specific robustness of dog as a model organism

**Fig. 4:**
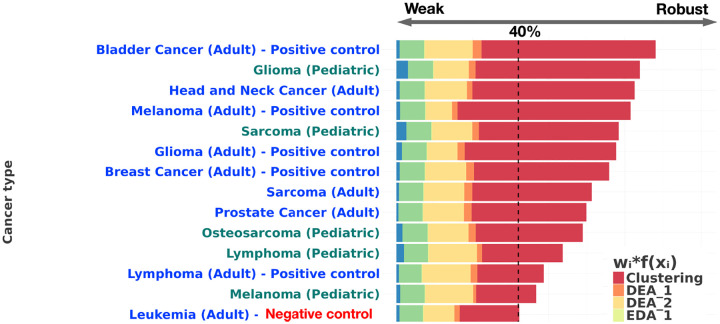
Multi-metric composite score evaluating dog cancers as models for adult and pediatric cancers. All dog cancers were assessed by a composite score integrating six features. Each stacked bar shows the score for a cancer type, scaled to 100%, with colors showing the contribution of each weighted feature to the overall score. The dashed vertical line separates cancer types from the LAML negative control.

Each previous analysis quantifies an aspect of tumor model robustness between humans and dogs. We integrated these into a combined formula to create a score of model robustness for each cancer type. Figure 4 and Supplemental Table 1 show cancer-specific scores, which ranged from 31% to 85% with the negative controls scoring 31% (ACC) and 40% (LAML). Adult cancers generally showed high concordance between species, whereas there is more score variability in the pediatric cancers, with scores ranging from 34% (ACC) to 80% (GBM/LGG). Formula feature weights (EDA_1 = 0.002, EDA_2 = 0.085, DEA_1 = 0.029, DEA_2 = 0.159, Clustering = 0.665, and ML = 0.060) vary significantly. While all features are score-driving, the Clustering score shows a notable increase in magnitude as the overall formula score increases.

### 3.5 High-scoring cancers have similar cross-species molecular differences

**Fig. 5:**
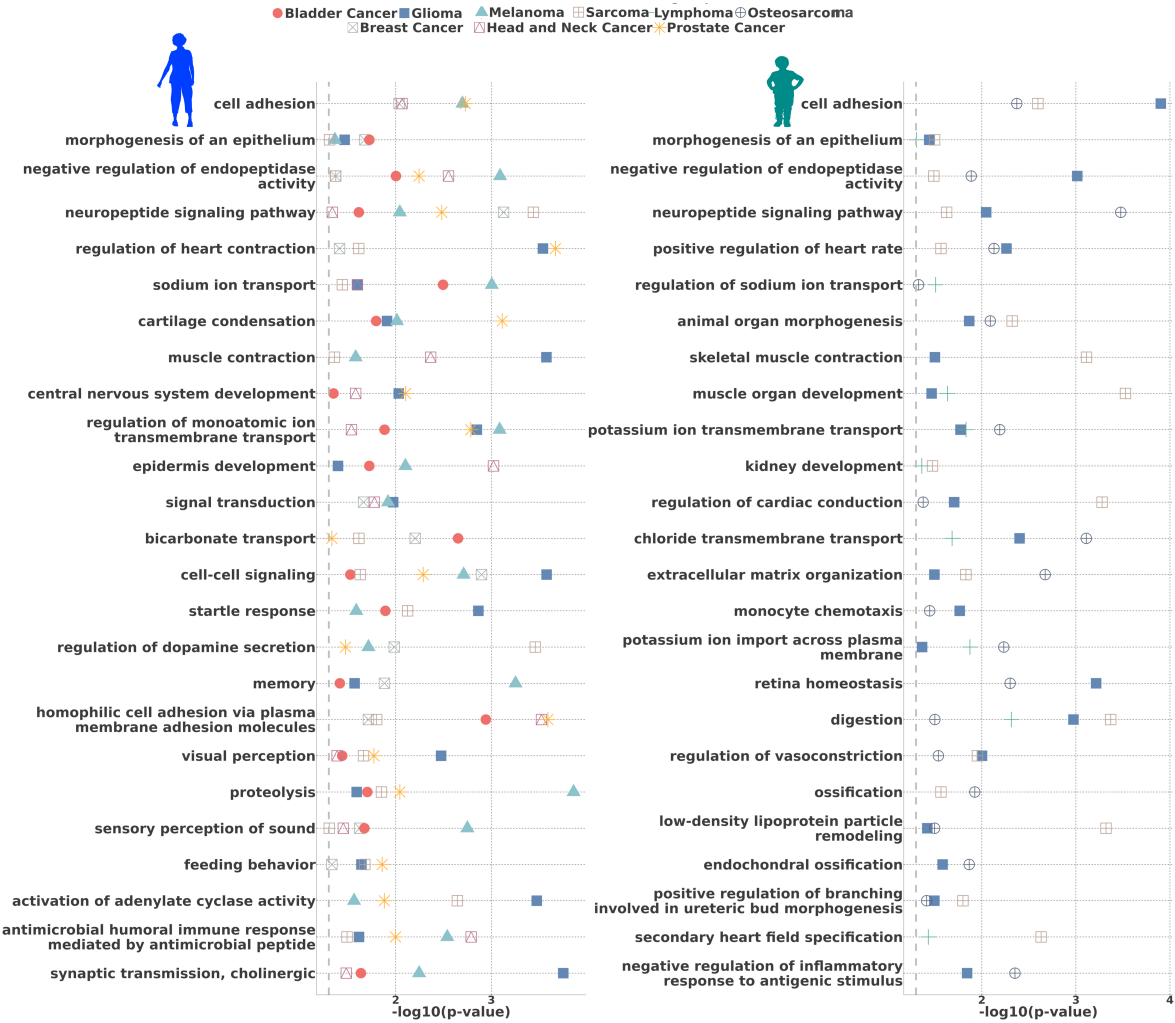
Biological processes that differ between human and dog samples across cancer types where dogs are strong models. We used GO BP enrichment of differentially expressed genes for each cancer type to identify recurring patterns across cancers. The top 25 GO BP terms most frequently enriched between dog and human cancers with scores above 50%, are shown separately for adult (left) and pediatric (right). The x-axis represents the enrichment significance as −log_10_(*P*-value), and each shape corresponds to a cancer type in which the biological process is significantly dysregulated between human and dog. The gray dashed line indicates the threshold of *P*-value = 0.05. GO terms are ordered between adult and pediatric panels based on semantic similarity using GOSemSim [87].

We next identified molecular patterns shared across the cancers for which the dog is a strong model. For this pancancer analysis, we consider cancers with high scores defined here as cancers scoring above 50%. To identify shared molecular features across these tumor types, we compared the overlap of differentially expressed GO BP between human and dog tumors (Figure 5a). In adult samples, 6 of 7 cancers have similar patterns of enriched GO terms (GO:0007218: neuropeptide signaling pathway, GO:0007267: cell-cell signaling, and GO:0010951: negative regulation of endopeptidase activity). In pediatric tumors, fewer terms are enriched across cancer types and only 1 GO term was enriched among all 4 high-scoring pediatric cancers (GO:0007586: digestion). Overall, shared GO term enrichment across cancers is more common in adult cancers than in pediatric cancers.

### 3.6 Organ-system tumor patterns are cross-species

**Fig. 6:**
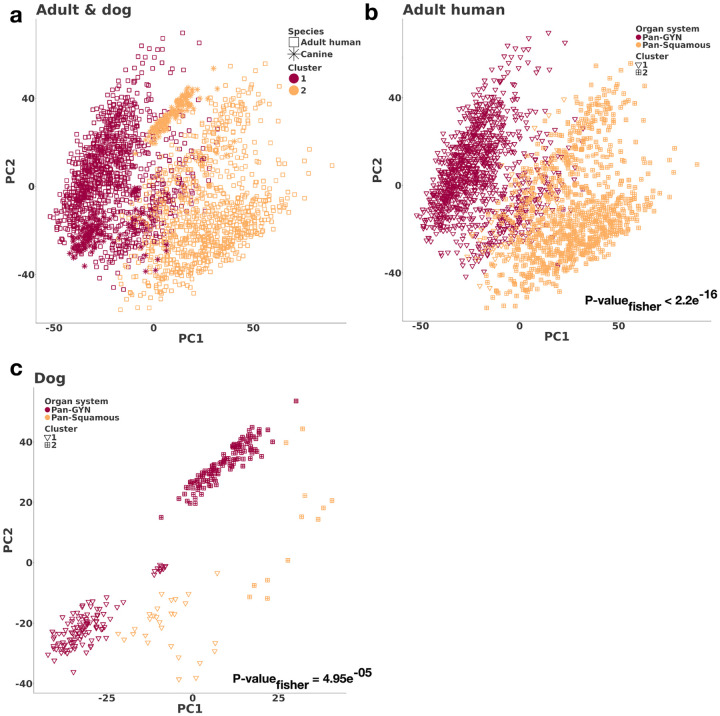
Multi-species pancancer clustering recapitulates adult-only results. (**a**) Hierarchical clustering of BRCA (pan-GYN), BLCA (pan-squamous) and HNSC (pan-squamous) from adult and dog tumors identified 2 clusters. (**b-c**) Show clustering results based on individual species and organ systems. The P-values indicate the association between cluster group and organ system.

To evaluate whether organ system groupings reported in the TCGA pancancer analysis [76, 89, 90] were also present in dogs, we applied repeated clustering with subsampling (see Methods) to the dog and human samples (Figure 6a). We observed that dog tumors (Figure 6c) are enriched for the human organ system groupings (Fisher’s exact test: Adult: BH adjusted *P*-value< 2.2 × 10^−16^, dog: BH adjusted *P*-value= 4.95 × 10^−05^, Figure 6b–c).

## 4 Discussion

Scientific understanding of cancer relies on studies across multiple species [2, 3, 5–8]. Despite this reliance, the field lacks systematic frameworks to evaluate cross-species molecular similarities across diverse tumor types [3]. Prior studies generally rely on differential gene expression analysis and derived metrics, which does not fully capture the spectrum of disease-specific molecular patterns shared between species [97, 98]. To address this gap, we create a quantitative formula for model robustness at the broader cancer type level. We apply this formula to a large pancancer dataset of human adult, human pediatric, and dog tumors to assess how well dog tumors reflect the transcriptomics landscape of adult and pediatric cancers. Our approach leverages multiple distinct but complementary transcriptomic analyses to comprehensively compare tumor molecular profiles across species.

We create a pancancer compendium of 5,875 RNA-seq samples from adult, pediatric, and dog tumors across 11 cancer types. Pairwise correlation analyses of samples in this compendium indicate that, across all cancers, the cancer type signal is stronger than species-specific differences (Figure 1b). Samples of the same cancer type across species show higher correlation than samples from different cancer types within the same species, with a mean difference of 0.1007 (95% CI: 0.10067–0.10081). This high level of similarity across cancers has also been observed in the context of mutated genes [14] and in single-cancer transcriptomics studies [22, 26, 31, 46, 73]. Furthermore, we found that samples largely cluster by cancer type (Figure 1d; one sided Wilcoxon rank sum test: *P*-value< 2.2 × 10^−16^). These results imply high levels of transcriptome-wide conservation between dog and human tumors, indicating that biologically meaningful cancer signals persist across species. Additionally as expected, we observed higher correlations within species than between species for the majority of cancers. However, for adult BLCA and adult/pediatric SARC, the opposite was true (Supplemental Figure 2a). We hypothesize that subtype heterogeneity may contribute to this pattern in SARC.

While overall transcriptome profiles are remarkably similar between dog and human samples from the same tumor type, species differences persist. To identify the functional roles of these transcriptomic differences and quantify their relevance to cancer, we analyzed the degree to which species-specific differences are cancer-related for each tumor type. In cases where fewer known cancer genes or dysregulated oncogenic pathways are differentially expressed, dog and human tumors show greater transcriptomic similarity. Adult BLCA is an excellent example of this. Differential expression analyses between dog and human tumors revealed that, for several cancers, a substantial fraction of dysregulated genes overlap with known cancer-associated genes and oncogenic pathways (Figure 2a–d). For example, OS and GBM/LGG in pediatric samples showed relatively strong enrichment of COSMIC cancer genes (one-sided binomial-test; BH adjusted GBM/LGG *P*-value = 2.70 × 10^−2^, OS *P*-value = 1.60 × 10^−2^). This enrichment suggests that dogs are not strong models for pediatric GBM/LGG or OS, despite prior studies reporting strong similarities between dogs and humans [46, 99–102], which highlights a limitation in relying solely on differential expression analysis to identify strong model organisms.

Given these limitations, which arise from the fact that a single metric only captures specific aspects of transcriptomic similarity, we developed a quantitative formula that integrates multiple features to quantify cancer-specific model robustness. Our formula integrates six features, each of which describes a unique but critical aspect of cancer-relatedness that can be derived from transcriptomic data. When applied to a multi-species pancancer dataset (Figure 4a), our formula shows that overall similarity is driven by the integration of all six features rather than by any single metric. Notably, we find that weighting recapitulates the level of analysis, with higher-level metrics having lower weights than more granular methods.

Scores were calculated for 11 cancer types, for which several have been studied previously. Overall, the cancer rankings derived using our formula match findings identified by previous studies in individual cancers not included among positive controls, such as pediatric GBM/LGG [46] and OS [20, 31], as well as adult HNSC [71, 72], SARC [25, 26], and PRAD [73], suggesting that our formula accurately quantifies overall model robustness for specific cancers. All positive controls scored above 70% with the exception of adult DLBC, however all still scored higher than the negative controls. We also provide scores for understudied dog cancer types, for which similarities can be inferred based on their relative positioning within the spectrum of cancers. When considering all cancers and given the variability in scores, we recommend caution in the use of dogs to model some of these human cancers. Lower-scoring cancers, such as pediatric SKCM, might be better suited for mechanistic studies rather than as a wholistic natural model. Overall, findings from our quantitative formula of model robustness highlight that researchers should prioritize dog models with consistently high composite scores for hypothesis testing and drug evaluation.

Among cancers with high model robustness scores, there are common patterns of biological processes that differ between species. Cross-species discrepancies are concentrated in broad pathway terms involved in cellular communication and proteolytic regulation, however these patterns diverge in the adult and pediatric cancers. Expression differences between species in high-scoring adult cancers are predominately related to neuropeptide signaling, cell-cell signaling and negative regulation of endopeptidase activity. In pediatric cancers, digestion related pathways were frequently different between dog and human tumors, suggesting that biological difference influence how tumors diverge between species.

These shared patterns of species-specific functional differences are also relevant to translational considerations. For example, prior studies have reported cross-species differences in drug absorption [103–105]. While not directly related to cancer, this does directly relate to disease management, and is an excellent example of the importance of One Health considerations. Our formula incorporates pathway and non-cancer biological processes, results from which can be used to determine the likely impacts of therapeutic response differences between species. Despite these differences, the analysis here highlights how cancer-related biological processes are largely conserved between humans and dogs, and that species differences are largely driven by factors unrelated or nominally related to cancer. While non-cancer processes may differ, warranting caution when evaluating the translational potential of dog models of cancer, the overarching functional conservation between tumors in the two species highlights the strength of the dog as a model of many human cancers. Researchers should carefully consider these areas of conservation and divergence when selecting endpoints for functional studies.

Given the overall cancer-type-specific similarities in adult cancers, we next evaluated organ-system level tumor clusters previously identified [76, 89, 90]. We found that dog tumors mirror the organ-system level structure previously seen in adult cancers associated with pan-GYN and pan-squamous lineages. This is observed in enrichment patterns for clusters and organ systems in both species separately (Fisher’s exact test: Adult: BH adjusted *P*-value< 2.2 × 10^−16^, dog: BH adjusted *P*-value= 4.95 × 10^−05^, Figure 6b–c). It suggests that dog tumors can recapitulate human organ-level pancancer structure, reinforcing their value for comparative genomics studies and for accelerating human cancer. Additionally, the presence of human clinical subtypes in naturally-occurring dog tumors provides a unique opportunity to explore the molecular drivers of these subtypes. We found that classifiers trained on adult cancer subtypes perform well in human and dog (Figure 3a). Notably, HNSC HPV- and BRCA LumA subtypes showed relatively strong subtype probabilities close to their human counterpart, with BRCA supported by previous studies [22, 94, 95].

While powerful, the formula presented here focuses on broader cancer-level similarities, which can obscure subtype-specific heterogeneity within a single cancer. To evaluate the degree to which this impacts scores for the cancers, we analyzed heterogeneity within and across species for two cancer types known for heterogeneity, GBM/LGG and SARC in both adult and pediatric. We observed significant differences in same-subtype and different-subtype groups, as the bootstrap 95% CI for the difference in mean correlations excluded zero in all comparisons; Supplemental Figure 6a. The mean differences were 0.046 (95% CI: 0.0460–0.0469) for adult GBM/LGG, 0.033 (95% CI: 0.0321–0.0341) for adult SARC, 0.060 (95% CI: 0.0594–0.0603) for pediatric GBM/LGG, and 0.024 (95% CI: 0.0226–0.0251) for pediatric SARC, highlighting heterogeneity that can be masked at this level of analysis. This is further complicated by inconsistencies in annotation granularity. The dog dataset used here contains relatively coarse cancer labels, whereas TCGA and Treehouse offer highly detailed classifications. These differences reflect not only varying annotation standards but also the evolution of the field over time, with more recent datasets incorporating increasingly fine-grained subtype definitions. When possible, we refine comparisons using shared subtypes. For instance, we separate OS from other SARC samples when data are available across both species. However, the relative scarcity of well established cross-species studies at fine-grained resolution across both species limits the availability of positive controls, motivating our focus on broader cancer-level comparisons. As future work, we plan to extend this framework to incorporate cancer subtypes.

Overall, we found that the dog is a robust model for many human cancer types, and that no single comparative analysis captures the full complexity of cancer-relatedness across species. We provide a data-driven approach for identifying which dog cancers are ideal for studying human cancers, which we apply to many cancers. Our results recapitulate previous findings for well-studied cancers and provide insights into other cancers where dog and human tumors have not been deeply comparatively evaluated. Results shown here highlight the importance of considering multiple complementary perspectives to obtain a more comprehensive view of cancer biology across species. These results represent a critical step towards the use of natural tumor animal models for preclinical studies, with the potential to extend this framework to many other mammalian species.

## 5 Conclusions

Our study presents a quantitative framework for assessing similarity between human and dog cancers. Using this framework, we found that dog cancers generally model adult tumors better than pediatric tumors, with a few exceptions. These findings help prioritize models for translational research and advance comparative oncology.

## Supplementary Material

1

## Figures and Tables

**Table 1:** Dog, adult, and pediatric cancer samples used in this study.

Cancer type	Total samples	Dog	Adult	Pediatric
Glioma	1,147	60 (5%)	671 (58%)	416 (36%)
Melanoma	557	106 (19%)	440 (79%)	11 (2%)
Sarcoma	491	43 (9%)	253 (51%)	195 (40%)
Adrenocortical Carcinoma	182	87 (48%)	75 (41%)	20 (11%)
Lymphoma	140	72 (51%)	48 (34%)	20 (14%)
Breast Cancer	1,414	332 (23%)	1,082 (76%)	-
Head and Neck Cancer	532	17 (3%)	515 (97%)	-
Prostate Cancer	517	24 (4%)	493 (95%)	-
Bladder Cancer	430	23 (5%)	407 (95%)	-
Osteosarcoma	277	133 (48%)	-	144 (52%)
Leukemia	188	16 (8%)	172 (91%)	-
**Total**	**5,875**	**913**	**4,156**	**806**

- indicates data are unavailable for that cancer type. Percentages represent the proportion of species-specific samples relative to the total number of samples.

## Data Availability

All code associated with this manuscript is available at https://github.com/GraimLab/dog_human_analysis. The results shown here are in whole or part based upon data generated by the TCGA Research Network: https://www.cancer.gov/tcga. Treehouse data are available under GEO accession GSE294351. Dog data is from Cahill et al. [5], which includes datasets from the following NCBI BioProject IDs: PRJNA557484, PRJNA579792, PRJNA671877, PRJEB34234, PRJNA260600, PRJNA559406, PRJNA339175, PRJNA308949, PRJNA421895, PRJNA421863, PRJNA475196, PRJNA389294, PRJNA527141, PRJNA749900, PRJNA203086, PRJNA385190, PRJNA841500, PRJNA489087, PRJNA763886, PRJNA561580, PRJNA691669, PRJNA598246, PRJNA884145, PRJNA628596, PRJNA376379, PRJNA267721, PRJNA797476, PRJEB47028, PRJNA492338, PRJNA803356, PRJNA680382, PRJNA615229, PRJNA689618, PRJNA345482, PRJNA353505, PRJNA820287, and PRJNA650547.

## Abbreviations

TCGA: The Cancer Genome Atlas
TMM: Trimmed mean of M-value
EDA: Exploratory data analysis
PCA: Principal component analysis
CI: Confidence intervals
DEG: Differentially expressed genes
GO: Gene Ontology
BP: Biological processes
COSMIC: Catalogue of Somatic Mutations in Cancer
MSigDB: Molecular signatures database
BH: Benjamini-Hochberg
CDF: Cumulative distribution function
BRCA: Breast cancer
GBM/LGG: Glioma
HNSC: Head and neck cancer
SARC: Sarcoma
BLCA: Bladder cancer
AUC: Area under the curve
M: Median gene expression
W: Wasserstein distance
LAML: Leukemia
ACC: Adrenocortical carcinoma
SKCM: Melanoma
DLBC: Lymphoma
OS: Osteosarcoma
PRAD: Prostate cancer
