# Supplementary material for: Leveraging Dog Models to Uncover Human Cancer Insights
Source: Res Sq. 2026 May 28:rs.3.rs-9783746. Preprint. [Version 1] doi: 10.21203/rs.3.rs-9783746/v1 (PMC13232445; doi:10.21203/rs.3.rs-9783746/v1)
Supplement: 1 [file NIHPPRS9783746V1-supplement-1.pdf]

# Supplemental Information

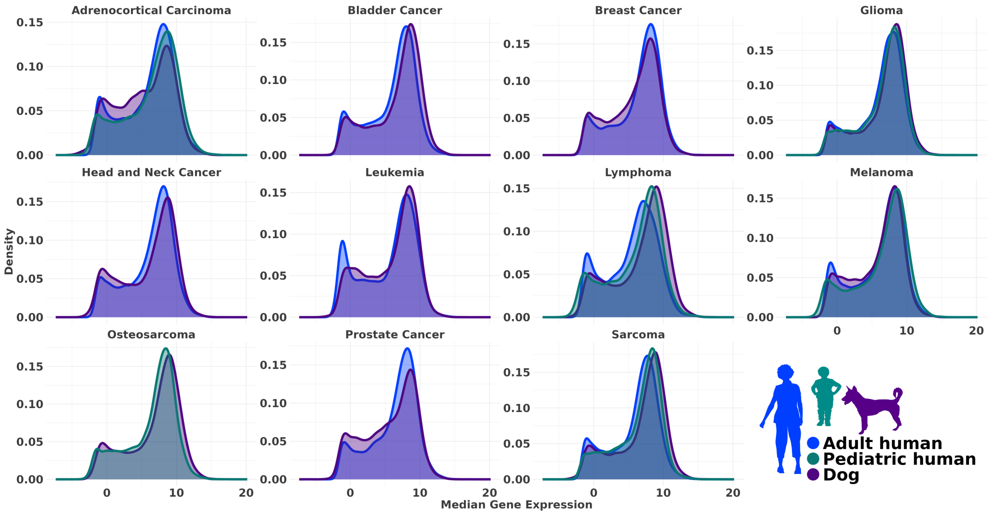


**Supplemental Figure 1**: **Median gene expression distributions between species.** Density plots show median expression per gene across adult, pediatric, and dog samples for individual cancer types. We quantified adult vs dog and pediatric vs dog median gene distributions using the normalized absolute distance and measured overall molecular-level similarity.

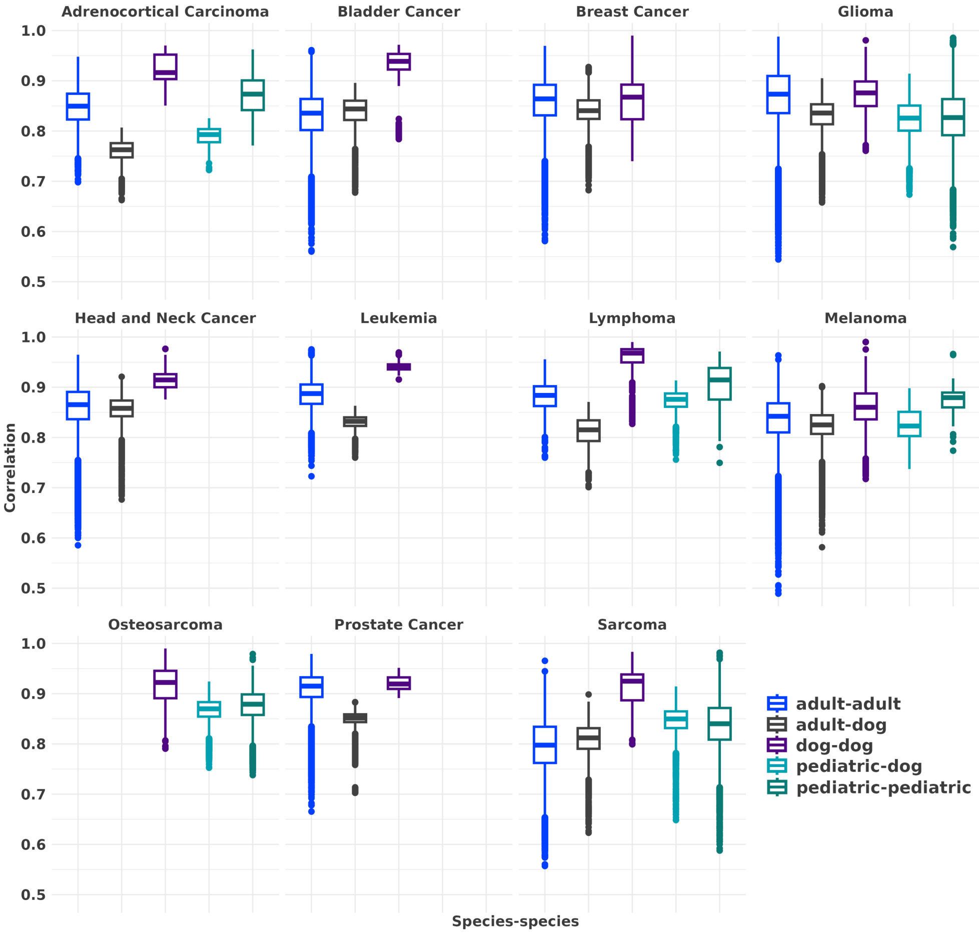


**Supplemental Figure 2**: **Intra-species and inter-species correlations per tumor type.** As illustrated in the box plots, we grouped sample pairs of the same cancer type into inter- and intra-species groupings. Adult BLCA and adult/pediatric SARC had higher mean inter-species correlations than intra-species correlations.

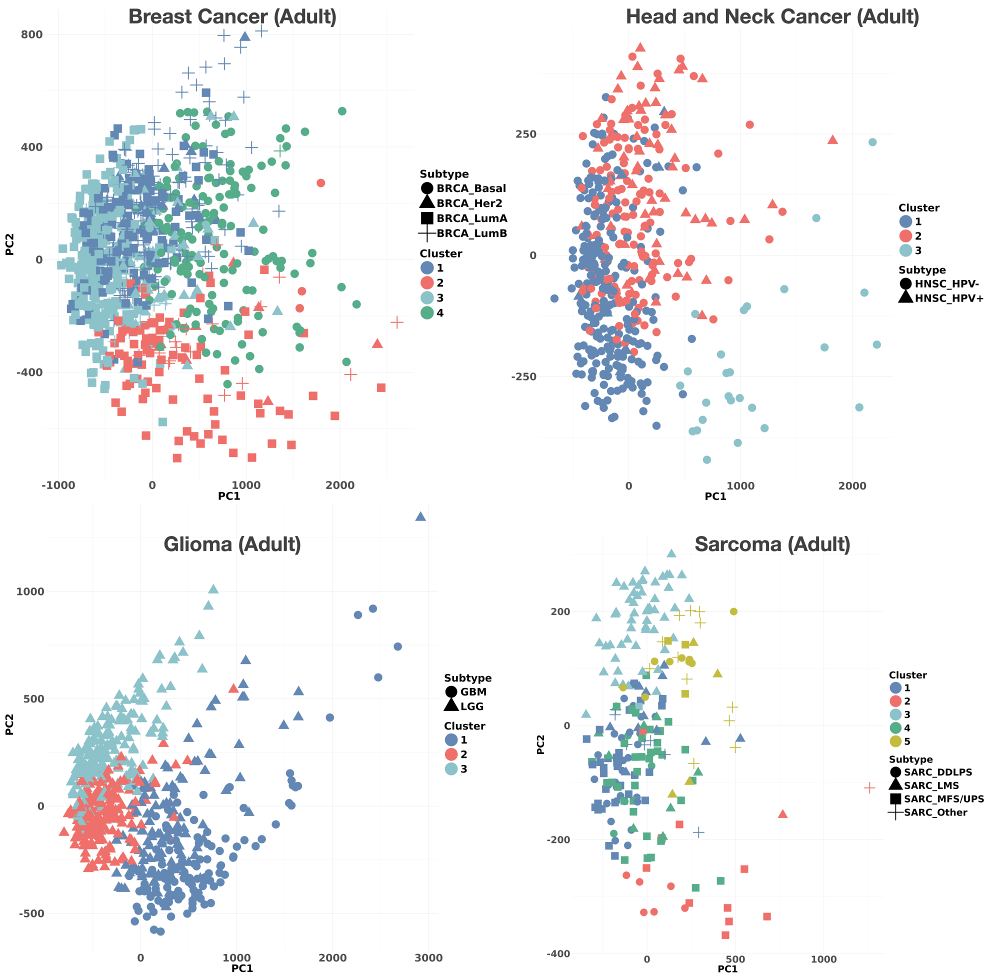


**Supplemental Figure 3**: **Adult-only clustering for four tumor types with TCGA subtypes.** The PCA plots show the adult-only clustering for BRCA, HNSC, GBM/LGG, and SARC. We found that adult-only clustering results statistically correlate with the TCGA subtypes (all four cancers Fisher's exact test: BH adjusted $P$-value $= 4.9975 \times 10^{-04}$).

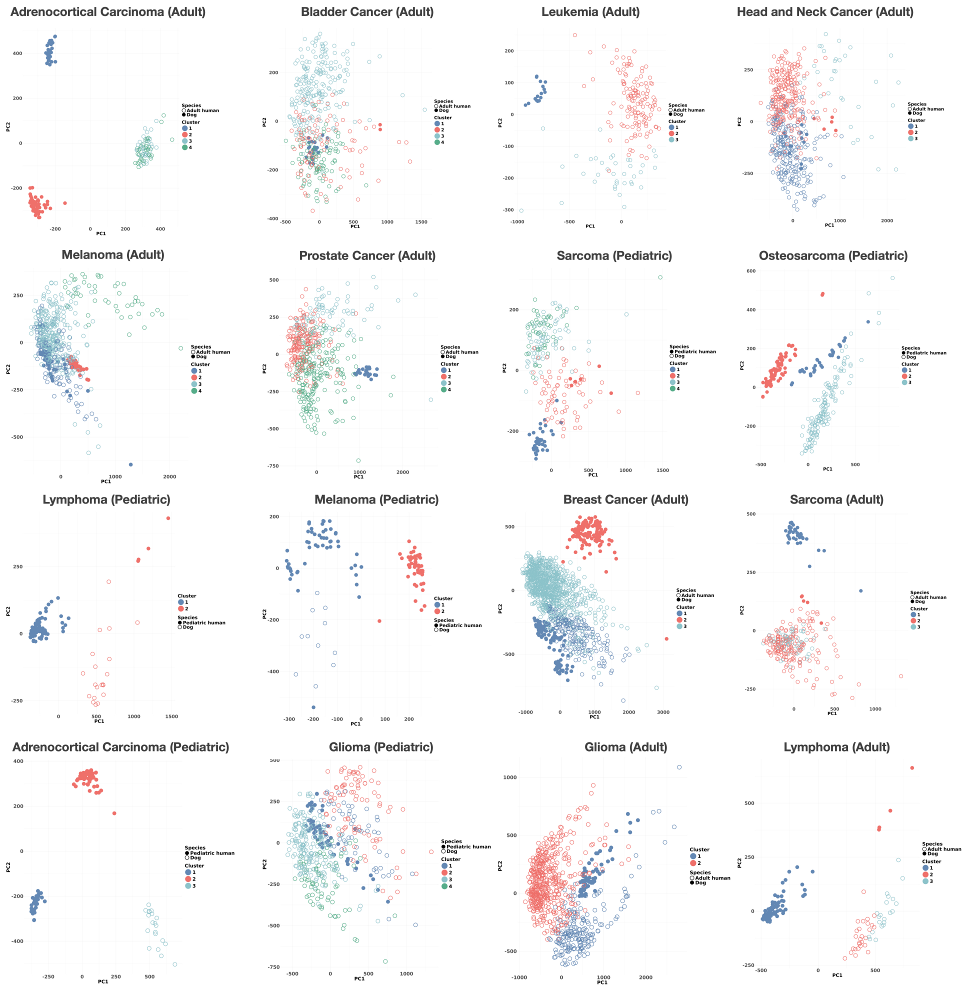


**Supplemental Figure 4**: **Combined species clustering for each tumor type.** The PCA plots show the combined species clustering (adult and dog, pediatric and dog). The minimum weighted centroid distance was calculated using clusters defined in the PC1-PC2 space to quantify species overlap.

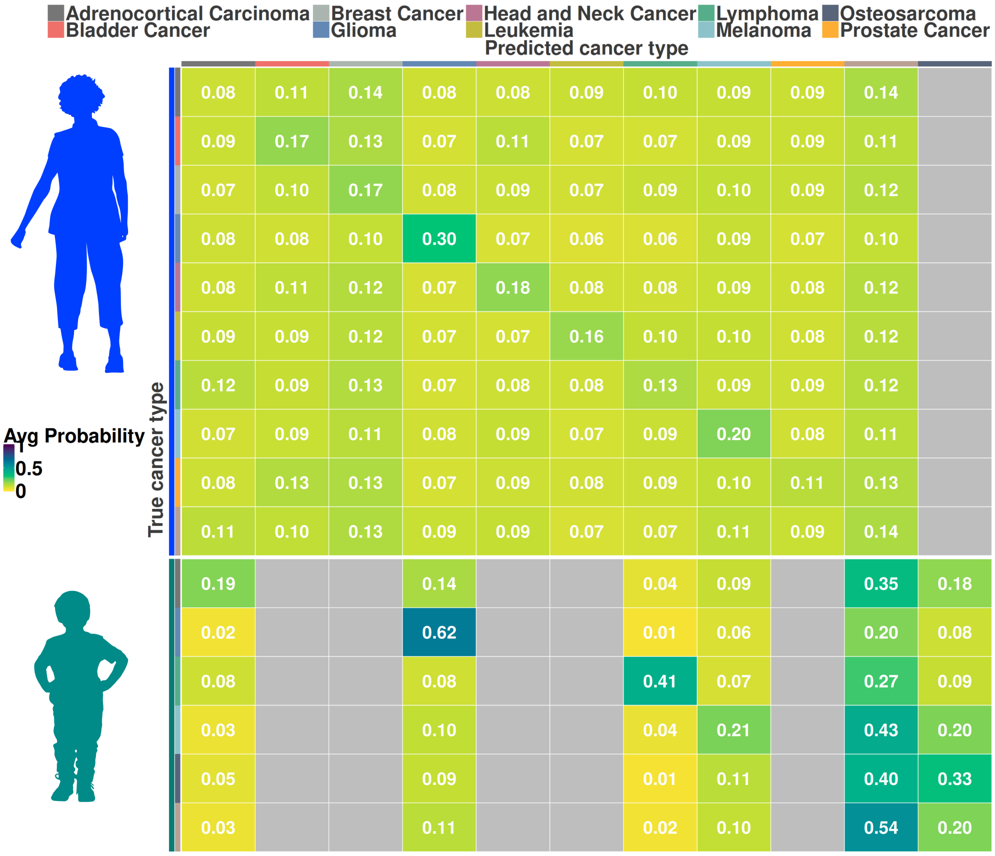


**Supplemental Figure 5**: **Cancer type prediction task of a human-trained model applied to dog.** The heatmap shows average predicted cancer type probabilities after training two multi-class classifiers on adult and pediatric data separately and applying them to dog samples. The y-axis represents the true cancer type label, and the x-axis shows the predicted classification labels.

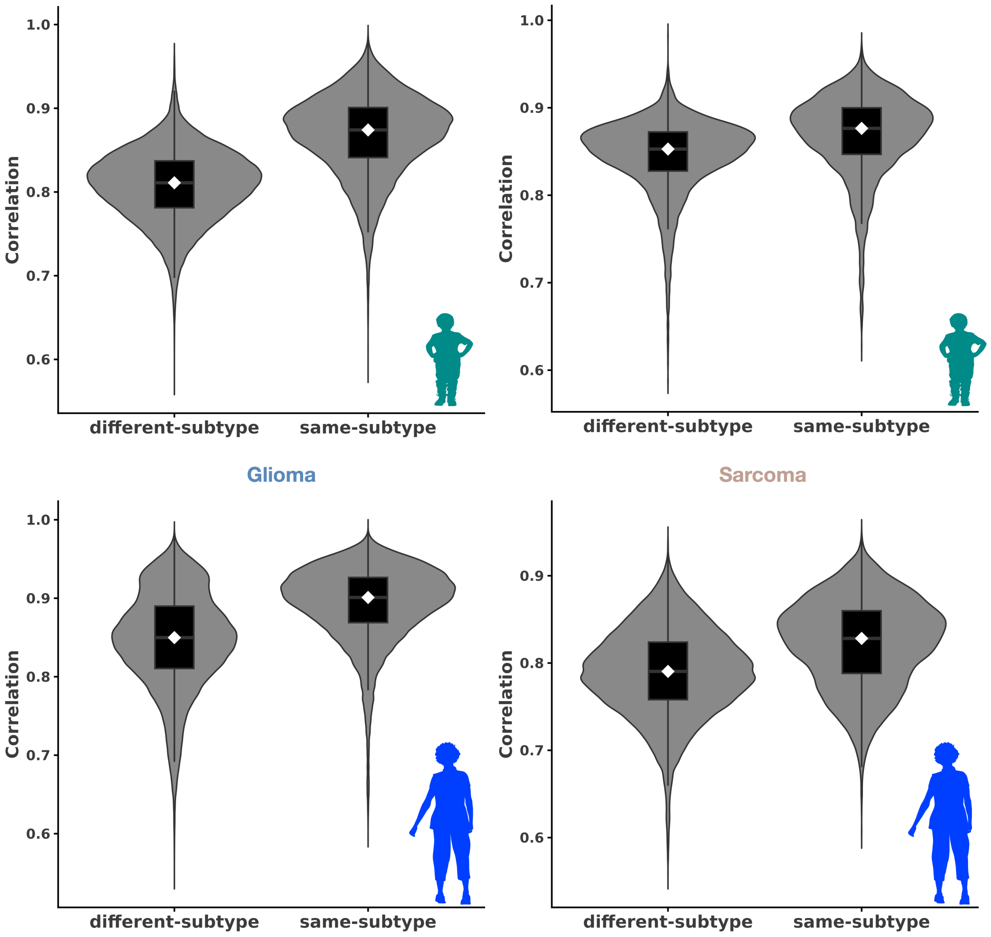


**Supplemental Figure 6**: **Subtype heterogeneity assessed using different-subtype and same-subtype correlations for GBM/LGG and SARC.** We assessed subtype heterogeneity in the data by comparing sample-sample correlation pairs within the same subtype and pairs from different subtypes at the broader cancer-level. For the comparisons, we included only subtypes having more than 10 samples.

**Supplemental Table 1**: Feature contributions for adult and pediatric cancers using the quantitative formula

| **Cancer type** | **EDA_1** | **EDA_2** | **DEA_1** | **DEA_2** | **Clustering** | **ML** | **Score** | **Human type** |
|---|---|---|---|---|---|---|---|---|
| Bladder Cancer - Positive control | 0.0216 | 0.9507 | 0.0000 | 0.0053 | 0.0756 | 0.1704 | 0.8505 | Adult |
| Glioma | 0.0091 | 0.9697 | 0.1389 | 0.1587 | 0.1040 | 0.6244 | 0.7988 | Pediatric |
| Head and Neck Cancer | 0.0183 | 0.9634 | 0.2308 | 0.0741 | 0.1105 | 0.1756 | 0.7820 | Adult |
| Melanoma - Positive control | 0.0068 | 0.9690 | 0.2381 | 0.2963 | 0.0784 | 0.1981 | 0.7685 | Adult |
| Sarcoma | 0.0158 | 0.9623 | 0.1538 | 0.0899 | 0.1831 | 0.5443 | 0.7295 | Pediatric |
| Glioma - Positive control | 0.0153 | 0.9554 | 0.1017 | 0.2328 | 0.1443 | 0.3018 | 0.7210 | Adult |
| Breast Cancer - Positive control | 0.0179 | 0.9707 | 0.0595 | 0.0847 | 0.2001 | 0.1734 | 0.6978 | Adult |
| Sarcoma | 0.0317 | 0.9425 | 0.0455 | 0.0899 | 0.2582 | 0.1354 | 0.6413 | Adult |
| Prostate Cancer | 0.0240 | 0.9282 | 0.0811 | 0.0847 | 0.2765 | 0.1148 | 0.6233 | Adult |
| Osteosarcoma | 0.0177 | 0.9653 | 0.1094 | 0.0899 | 0.3086 | 0.3321 | 0.6113 | Pediatric |
| Lymphoma | 0.0309 | 0.9295 | 0.2727 | 0.0000 | 0.4302 | 0.4076 | 0.5458 | Pediatric |
| Lymphoma - Positive control | 0.0471 | 0.8798 | 0.1404 | 0.0000 | 0.5062 | 0.1323 | 0.4833 | Adult |
| Melanoma | 0.0212 | 0.9445 | 0.5000 | 0.0053 | 0.5425 | 0.2060 | 0.4588 | Pediatric |
| Leukemia - Negative control | 0.0153 | 0.9099 | 0.2464 | 0.2222 | 0.5460 | 0.1641 | 0.4031 | Adult |
| Adrenocortical Carcinoma | 0.0355 | 0.8836 | 0.0000 | 0.0847 | 0.7762 | 0.1934 | 0.3356 | Pediatric |
| Adrenocortical Carcinoma - Negative control | 0.0223 | 0.8619 | 0.0108 | 0.1164 | 0.7935 | 0.0840 | 0.3110 | Adult |

Feature values are reported prior to transformation, and human type indicates whether the dog comparison corresponds to adult or pediatric.

**Supplemental Table 2**: Bootstrap 95% confidence intervals for pairwise group differences across cancer types

| Cancer type | Comparison | Mean diff | 95% CI |
|---|---|---|---|
| Glioma | adult-adult vs adult-dog | 0.03699 | (0.03667, 0.03736) |
| Glioma | adult-dog vs dog-dog | -0.04305 | (-0.04437, -0.04185) |
| Glioma | pediatric-dog vs pediatric-pediatric | -0.00357 | (-0.00410, -0.00308) |
| Glioma | dog-dog vs pediatric-dog | 0.05005 | (0.04870, 0.05132) |
| Head and Neck Cancer | adult-adult vs adult-dog | 0.00454 | (0.00391, 0.00511) |
| Head and Neck Cancer | adult-dog vs dog-dog | -0.05899 | (-0.06156, -0.05642) |
| Bladder Cancer | adult-adult vs adult-dog | -0.00826 | (-0.00891, -0.00763) |
| Bladder Cancer | adult-dog vs dog-dog | -0.08132 | (-0.08615, -0.07629) |
| Melanoma | adult-adult vs adult-dog | 0.01134 | (0.01098, 0.01166) |
| Melanoma | adult-dog vs dog-dog | -0.03619 | (-0.03690, -0.03550) |
| Melanoma | pediatric-dog vs pediatric-pediatric | -0.04731 | (-0.05418, -0.04079) |
| Melanoma | dog-dog vs pediatric-dog | 0.03386 | (0.03205, 0.03578) |
| Breast Cancer | adult-adult vs adult-dog | 0.01625 | (0.01612, 0.01637) |
| Breast Cancer | adult-dog vs dog-dog | -0.01846 | (-0.01872, -0.01819) |
| Sarcoma | adult-adult vs adult-dog | -0.01191 | (-0.01264, -0.01118) |
| Sarcoma | adult-dog vs dog-dog | -0.10704 | (-0.10868, -0.10529) |
| Sarcoma | pediatric-dog vs pediatric-pediatric | 0.00765 | (0.00683, 0.00843) |
| Sarcoma | dog-dog vs pediatric-dog | 0.06992 | (0.06817, 0.07171) |
| Lymphoma | adult-adult vs adult-dog | 0.06949 | (0.06791, 0.07108) |
| Lymphoma | adult-dog vs dog-dog | -0.14137 | (-0.14269, -0.14002) |
| Lymphoma | pediatric-dog vs pediatric-pediatric | -0.03226 | (-0.03682, -0.02746) |
| Lymphoma | dog-dog vs pediatric-dog | 0.08332 | (0.08163, 0.08493) |
| Prostate Cancer | adult-adult vs adult-dog | 0.06016 | (0.05986, 0.06047) |
| Prostate Cancer | adult-dog vs dog-dog | -0.07112 | (-0.07235, -0.06985) |
| Leukemia | adult-adult vs adult-dog | 0.05483 | (0.05423, 0.05542) |
| Leukemia | adult-dog vs dog-dog | -0.11037 | (-0.11162, -0.10911) |
| Adrenocortical Carcinoma | adult-adult vs adult-dog | 0.08531 | (0.08405, 0.08650) |
| Adrenocortical Carcinoma | adult-dog vs dog-dog | -0.16557 | (-0.16635, -0.16473) |
| Adrenocortical Carcinoma | pediatric-dog vs pediatric-pediatric | -0.07975 | (-0.08411, -0.07532) |
| Adrenocortical Carcinoma | dog-dog vs pediatric-dog | 0.13580 | (0.13472, 0.13693) |
| Osteosarcoma | pediatric-dog vs pediatric-pediatric | -0.00882 | (-0.00941, -0.00826) |
| Osteosarcoma | dog-dog vs pediatric-dog | 0.05004 | (0.04943, 0.05065) |

Bootstrap resampling (1,000 replicates, percentile method) was used to estimate mean differences (mean diff) and construct 95% confidence intervals for differences in mean correlations between inter- and intra-species groups.

**Supplemental Table 3**: Harmonized cancer types between human and dog

| Adult cancer type | Pediatric cancer type | Dog cancer type | Harmonized cancer type |
|---|---|---|---|
| Astrocytoma<br>Glioblastoma multiforme<br>Low-grade glioma (NOS)<br>Oligoastrocytoma<br>Oligodendroglioma | Atypical teratoid/rhabdoid tumor<br>Choroid plexus carcinoma<br>Embryonal tumor with multilayered rosettes<br>Ependymoma<br>Ganglioglioma<br>Glioma<br>Gliomatosis cerebri<br>Gliosarcoma<br>Medulloblastoma<br>Neuroepithelial tumor<br>Pineal parenchymal tumor<br>Supratentorial embryonal tumor NOS | Glioma | Glioma |
| Dedifferentiated liposarcoma<br>Desmoid/aggressive fibromatosis<br>Leiomyosarcoma<br>Malignant peripheral nerve sheath tumor<br>Myxofibrosarcoma<br>Synovial sarcoma<br>undifferentiated pleomorphic sarcoma/malignant fibrous histiocytoma/high-grade spindle cell sarcoma | Alveolar rhabdomyosarcoma<br>Alveolar soft part sarcoma<br>CIC rearranged sarcoma<br>Desmoplastic small round cell tumor<br>Embryonal rhabdomyosarcoma<br>Epithelioid sarcoma<br>Infantile fibrosarcoma<br>INI-deficient soft tissue sarcoma NOS<br>Malignant peripheral nerve sheath tumor<br>Rhabdomyosarcoma<br>Sarcoma<br>Sclerosing epithelioid fibrosarcoma<br>Spindle cell/sclerosing rhabdomyosarcoma<br>Synovial sarcoma<br>Undifferentiated pleomorphic sarcoma<br>Undifferentiated sarcoma NOS | Soft tissue sarcoma | Sarcoma |
| - | Osteosarcoma | Osteosarcoma | Osteosarcoma |
| Cutaneous melanoma | Melanoma<br>Skin cutaneous melanoma | Acral melanoma<br>Cutaneous melanoma<br>Mucosal melanoma<br>Oral melanoma | Melanoma |
| Acute myeloid leukemia | - | Mast cell tumor | Leukemia |
| Diffuse large B-cell lymphoma, NOS | Lymphoma | B-Cell lymphoma | Lymphoma |
| Breast invasive carcinoma (NOS)<br>Breast invasive ductal carcinoma<br>Breast invasive lobular carcinoma<br>Breast invasive mixed mucinous carcinoma<br>Invasive breast carcinoma<br>Metaplastic breast cancer | - | Mammary tumor | Breast Cancer |
| Adrenocortical carcinoma | Adrenocortical carcinoma<br>Adrenocortical cancer | Adrenal tumor | Adrenocortical Carcinoma |
| Head and neck squamous cell carcinoma | - | Head and Neck Squamous Carcinoma | Head and Neck Cancer |
| Prostate adenocarcinoma | - | Prostate cancer | Prostate Cancer |
| Bladder urothelial carcinoma | - | Bladder tumor | Bladder Cancer |

- indicates data are unavailable or not sufficient. Pediatric leukemia samples were not included in the analysis, as only adult leukemia samples were used as the negative control.
